# Intraindividual comparison of [^68^ Ga]-Ga-PSMA-11 and [^18^F]-F-PSMA-1007 in prostate cancer patients: a retrospective single-center analysis

**DOI:** 10.1186/s13550-021-00845-z

**Published:** 2021-10-19

**Authors:** Sebastian Hoberück, Steffen Löck, Angelika Borkowetz, Ulrich Sommer, Robert Winzer, Klaus Zöphel, Dieter Fedders, Enrico Michler, Jörg Kotzerke, Klaus Kopka, Tobias Hölscher, Anja Braune

**Affiliations:** 1 Department of Nuclear Medicine, Faculty of Medicine and University Hospital Carl Gustav Carus, TU Dresden, Fetscherstr. 74, 01307 Dresden, Germany; 2 Department of Nuclear Medicine, Helios Klinikum Erfurt, Erfurt, Germany; 3 OncoRay – National Center for Radiation Research in Oncology, Faculty of Medicine and University Hospital Carl Gustav Carus, TU Dresden, Helmholtz-Zentrum Dresden - Rossendorf, Dresden, Germany; 4 Department of Urology, Faculty of Medicine and University Hospital Carl Gustav Carus, TU Dresden, Dresden, Germany; 5 Department of Pathology, Faculty of Medicine and University Hospital Carl Gustav Carus, TU Dresden, Dresden, Germany; 6 Department of Radiology, Faculty of Medicine and University Hospital Carl Gustav Carus, TU Dresden, Dresden, Germany; 7 Department of Nuclear Medicine, Klinikum Chemnitz gGmbH, Chemnitz, Germany; 8 Institute of Radiopharmaceutical Cancer Research, Helmholtz-Zentrum Dresden-Rossendorf (HZDR), Dresden, Dresden, Germany; 9 Faculty of Chemistry and Food Chemistry, School of Science, TU Dresden, Dresden, Germany; 10 Department of Radiotherapy and Radiation Oncology, Faculty of Medicine and University Hospital Carl Gustav Carus, TU Dresden, Dresden, Germany

**Keywords:** PSMA, Prostate cancer, PET, [^18^F]-F-PSMA-1007, [^68^Ga]-Ga-PSMA-11, miTNM

## Abstract

**Background:**

The analysis aimed to compare the radiotracers [^68^Ga]-Ga-PSMA-11 and [^18^F]-F-PSMA-1007 intraindividually in terms of malignant lesions, mi(molecular-imaging)TNM staging and presumable unspecific lesions retrospectively as used in routine clinical practice.

**Methods:**

A retrospective analysis of 46 prostate cancer patients (median age: 71 years) who underwent consecutive [^68^Ga]-Ga-PSMA-11- and [^18^F]-F-PSMA-1007-PET/CT or PET/MRI within a mean of 12 ± 8.0 days was performed. MiTNM staging was performed in both studies by two nuclear medicine physicians who were blinded to the results of the other tracer. After intradisciplinary and interdisciplinary consensus with two radiologists was reached, differences in both malignant and presumable nonspecific tracer accumulation were analyzed.

**Results:**

Differences in terms of miTNM stages in both studies occurred in nine of the 46 patients (19.6%). The miT stages differed in five patients (10.9%), the miN stages differed in three patients (6.5%), and different miM stages occurred only in one patient who was upstaged in [^18^F]-F-PSMA-1007 PET. Concordant miTNM stages were obtained in 37 patients (80.4%). There was no significant difference between [^18^F]-F-PSMA-1007 and [^68^Ga]-Ga-PSMA-11 in the SUV_max_ locally (31.5 vs. 32.7; *p* = 0.658), in lymph node metastases (28.9 vs. 24.9; *p* = 0.30) or in bone metastases (22.9 vs. 27.6; *p* = 0.286). In [^18^F]-F-PSMA-1007 PET, more patients featured presumable unspecific uptake in the lymph nodes (52.2% vs. 28.3%; *p*: < 0.001), bones (71.7% vs. 23.9%; *p* < 0.001) and ganglia (71.7% vs. 43.5%; *p* < 0.001). Probable unspecific, exclusively [^18^F]-F-PSMA-1007-positive lesions mainly occurred in the ribs (58.7%), axillary lymph nodes (39.1%) and cervical ganglia (28.3%).

**Conclusion:**

In terms of miTNM staging, both tracers appeared widely exchangeable, as no tracer relevantly outperformed the other. The differences between the two tracers were far more common in presumable unspecific lesions than in malignant spots. A routinely performed two-tracer study could not be shown to be superior. Since it seems at least challenging for most nuclear medicine departments to provide both [^18^F]-F-PSMA-1007 and [^68^Ga]-Ga-PSMA-11, it appears reasonable to choose the PSMA radiotracer depending on local availability with attention to the greater occurrence of nonspecific bone findings with [^18^F]-F-PSMA-1007.

## Background

Prostate cancer (PCa) is the world’s most common cancer in men [1]. Within the variety of radiolabeled PSMA ligands [2, 3], [^68^Ga]-Ga-labeled PSMA ligands have become state of the art in molecular imaging of PCa in primary and recurrent diseases, as well as in therapy monitoring [4–9].

However, as in all [^68^Ga]-Ga-labeled radiotracers, the capacity of the examination of [^68^Ga]-Ga-PSMA-11 is limited by its short half-life of only 68min, requiring in most cases in-house production and a sufficient generator supply. However, cyclotron-based production methods have recently been developed [10]. The output of more than 100 GBq could possibly allow satellite distribution of [^68^Ga]-Ga-labeled tracers equivalently to [^18^F]-F-tracers. Nevertheless, the commercial availability of [^68^Ga]-Ga/[^68^Ge]-Ga-generators allows cyclotron independent tracer production for institutions, that have no such access.

The implementation of [^18^F]-F-PSMA ligands may overcome the limitations of the short half-life of [^68^Ga]-Ga. Furthermore, the end point positron energy of [^18^F]-F is much lower than that of [^68^Ga]-Ga (0.65 vs. 1.90 MeV), which reduces the positron range in tissue and may improve spatial resolution [11]. The different physical properties of the nuclides may influence the SUV values of [^18^F]-F-PSMA-1007 and [^68^Ga]-Ga-PSMA-11.

Biokinetically, [^18^F]-F-PSMA-1007 features a lower urinary excretion than [^68^Ga]-Ga-PSMA-11 [8] and other ^68^Ga-labeled PSMA ligands [12, 13], which potentially improves the detectability of local recurrences [14].

Despite their dissimilar biokinetics, [^68^Ga]-Ga- and [^18^F]-F-PSMA-ligands in general [15] and [^18^F]-F-PSMA-1007 [14] vs. [^68^Ga]-Ga-PSMA-11 specifically are considered widely exchangeable for most indications. However, there are only a few clinical studies directly comparing [^68^Ga]-Ga-PSMA-11 and [^18^F]-F-PSMA-1007. Matched comparisons in patients with biochemical recurrence (BCR) [16] and intraindividual comparisons in therapy-naive patients [17] have shown widely corresponding results in terms of malignant results, while [^18^F]-F-PSMA-1007 has indicated more nonspecific lesions [16]. Our analysis aimed to identify specific clinical situations in which one tracer outperforms the other and to assess if there is a relevant incremental value of dual tracer studies.

## Material and methods

### Patients

**Fig. 1:**
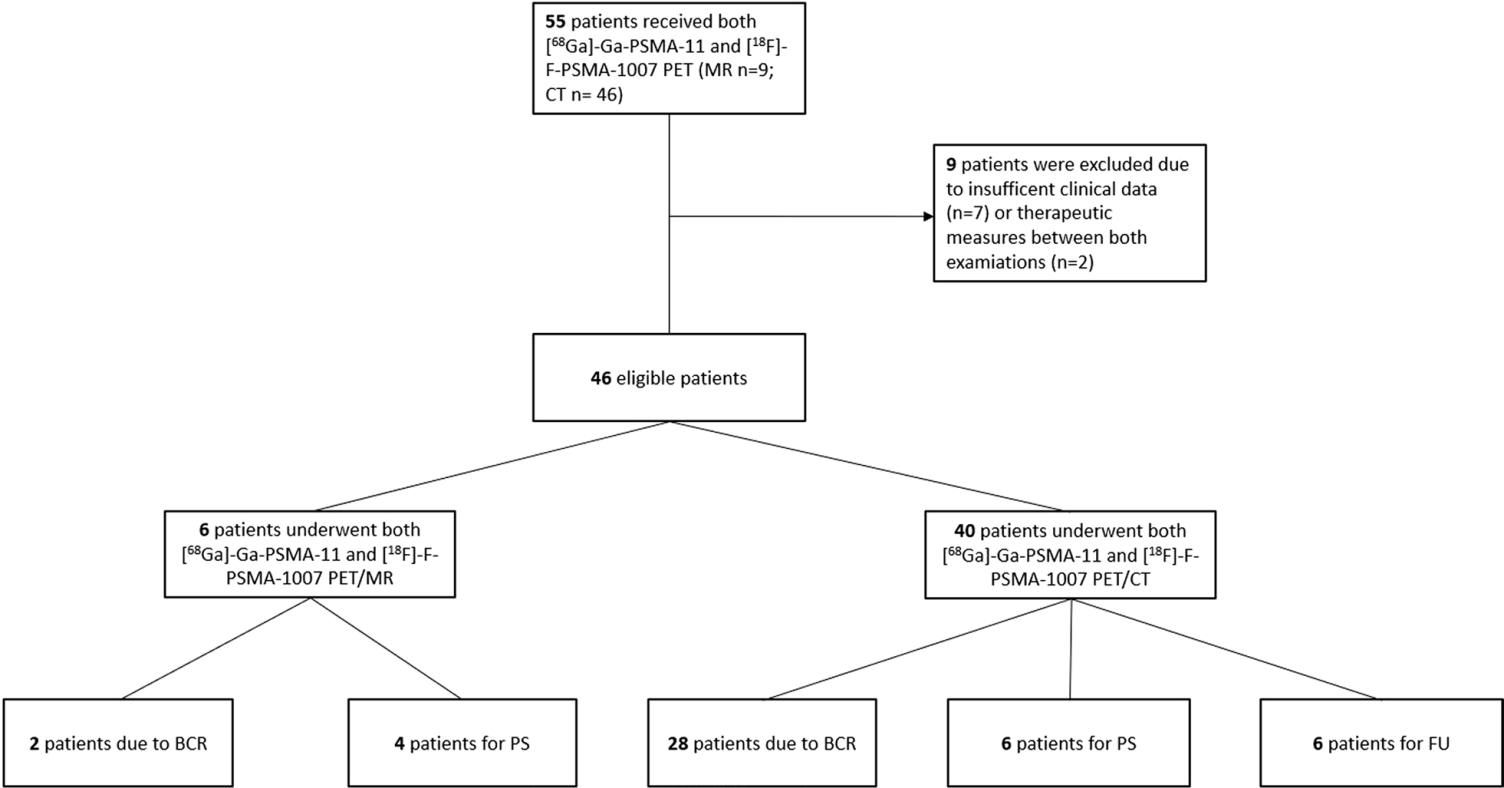
Patients who received both [^18^F]-F-PSMA-1007 and [^68^Ga]-Ga-PSMA-11 by indication and device. BCR: Biochemical recurrence; PS: primary staging; FU: follow-up of pre-known metastases

Between 07/20 and 12/20, fifty-five prostate cancer patients underwent both [^68^Ga]-Ga-PSMA-11 and [^18^F]-F-PSMA-1007 PET/CT or PET/MRI. Two patients received therapeutic measures between the two studies and were therefore not eligible for analysis. Seven more patients were excluded due to a lack of clinical data, no interdisciplinary tumor board presentation or missing consent for scientific re-evaluation (Fig. 1). The remaining 46 patients underwent both examinations for different clinical indications within a mean of 12 ± 8.0 days. All patients underwent both examinations on the same device. One patient underwent [^18^F]-F-PSMA-1007 PET before [^68^Ga]-Ga-PSMA-11 PET (13 days). All other patients received [^68^Ga]-Ga-PSMA-11 PET first. All 46 patients in this retrospective single-center analysis were discussed by an interdisciplinary tumor board, and an interdisciplinary consensus of all imaging results and further therapeutic management were decided upon. Written informed consent was obtained from all patients for the clinically indicated examination and the consecutive scientific analysis of their clinical and imaging data. The institutional review board of the local ethics committee at our medical faculty approved this study.

#### Radiotracer preparation

The radiotracers [^68^Ga]-Ga-PSMA-11 [18] and [^18^F]-F-PSMA-1007 [19] were synthesized as previously described.

#### Imaging protocol

In addition to hydration with at least 1.5 L of water, no specific patient preparations were required for either PET examination. As per clinical routine, no diuretics were administered.

For the [^68^Ga]-Ga-PSMA-11 examinations, a median of 149 MBq (range: 111–161 MBq) was intravenously injected, and acquisition started after a median of 106 min p.i. (mean: 110 ± 18 min; range: 90–182 min), while for [^18^F]-F-PSMA-1007 PET, a nearly equal median of 154 MBq (range: 123–175 MBq) was applied, and imaging started after a median of 103 min p.i. (mean: 104 ± 11 min; range: 90–128 min).

The PET/CT scans of 40 patients were acquired using a Biograph Vision 600 device (Siemens Healthineers, Knoxville, USA). A low-dose CT scan was acquired from the whole body (CARE Dose 4D with a reference of 11 mAs and 12 kV, spiral pitch factor of 1.5, 3.0 mm slice thickness) and used for attenuation correction of the following PET scan. The emission PET scan was obtained using continuous bed motion with a speed of 2.2 mm/s for [^18^F]-F-PSMA-1007 and 1.4 mm/s for [^68^Ga]-Ga-PSMA scans.

The PET/MRI scans of six patients were acquired using a 3 Tesla Ingenuity TOF PET/MRI scanner (Philips Medical Systems, Best, Netherlands). Ten to eleven bed positions were acquired with a scan time of 3 min each. For attenuation correction, a T1-weighted gradient echo scan with 4.1 ms/2.3 ms (repetition time/echo time), a field of view of 600 × 600 mm, and a slice thickness of 6 mm was performed.

### Imaging reconstruction

PET images were reconstructed using an ordered subset expectation maximization 3D iterative reconstruction with four iterations and five subsets, applying point spread function (PSF), time of flight (ToF), and correction for attenuation and scatter without postfiltering. The resulting PET images had an image matrix size of 440 × 440 and a voxel size of 1.65 × 1.65 × 3.0 mm^3^.

PET/MR images were reconstructed using a BLOB-OS-TF algorithm with MRI-based attenuation correction (attenuation map with 3 biological classes: air, lungs, soft tissue). All PET/MR images had an image matrix size of 144 × 144 and a voxel size of 4.0 × 4.0 × 4.0 mm^3^.

### Image analysis

Two nuclear medicine physicians (SH, EM) and two radiologists (RW, DF), both experienced in PSMA PET reporting and blinded to the results of the other examination, used Syngo.via Software (VB30a, Siemens Healthineers, Erlangen, Germany) to determine pathological uptake and to identify the reference lesions. Intra- and interdisciplinary consensus was reached in case of diverging results. In terms of bone lesions, the differentiation between presumably nonspecific and metastatic lesions was made in consensus, taking into account the intensity of tracer accumulation (miPSMA), lesion size and morphologic appearance. If there was a morphologic correlate, a lower miPSMA score of just 1 was sufficient for a lesion to be rated as malignant; otherwise, a score of 2 was considered suggestive of malignancy [20].

In the primarily performed visual analysis, pathological uptake was initially assumed if lesions visually showed tracer uptake higher than the local background [21]. Depending on the localization, they were designated as either local (prostate) tumors, (extra) pelvic lymphonodal lesions or distant metastases. Typical benign tracer accumulations [22] were captured separately. Each patient was staged using the miTNM classification [20]. In miT staging, a distinction was made between miT0 (absence of any tumor), miT2 for organ-confined tumors that were either unifocal (miT2u) or multifocal (miT2m), miT3 for non-organ-confined tumors with extracapsular extension (miT3a) or seminal vesicle invasion (miT3b) and miT4 for tumor invasion into an area other than the seminal vesicle. miTr is an extra stage for local recurrence after radical prostatectomy. MiT1 is not defined. In lymph node staging, a separation is made between the absence of any metastasis (miN0) and the effect of only one (miN1) or more than one (miN2) pelvic lymph node region [23].

Extrapelvic lymph node metastases were rated as miT1a. miM staging further separates the presence of bone metastases (miT1b) and all other metastases (miT1c).

The most intense lesion of every pelvic and extrapelvic lymphonodal region and distant metastatic region was scored according to the miPSMA expression score [20], and the SUV_max_ and SUV_peak_ were acquired. The score ranged from 0 (uptake < blood pool) to 3 (uptake ≥ parotid gland). It was determined based on the SUV_mean_ of both the lesions and the reference regions, which included the liver in [^68^Ga]-Ga-PSMA-11 studies and the spleen in studies with biliary excreted [^18^F]-F-PSMA-1007. If a lesion was not separable from the local background at one time point, it was scored as 0, regardless of its SUV_mean_. In this case, it was excluded from any further analysis. Sufficiently large [20] VOIs were inserted in the following reference regions: liver (3 cm diameter), spleen, thoracic aorta (2 cm diameter) and parotid glands (1.5 cm diameter), and the SUV_max_ and SUV_mean_ values were determined. For the parotid glands, the values were averaged.

Furthermore, the quantity of all pathologic lesions in every region was determined semiquantitatively by categorizing the uptake pattern of lymphatic and distant metastatic regions separately and in total in unifocal (*n* = 1), oligofocal (2–3) and multifocal/disseminated (*n* > 3).

For subsequent quantitative analysis, sufficiently large volumes of interest (VOIs) of each pathological lesion were defined to cover the whole lesion to obtain the SUV_max_ and SUV_peak_ of each lesion.

Analogously, the nonspecific tracer accumulations were compared by analyzing the pattern and PSMA ligand accumulation in all predilection spots.

### Statistical analysis

For descriptive analyses, normally distributed metric parameters were evaluated by the mean and standard deviation, and skewed metric parameters were evaluated by the median and range. The Shapiro–Wilk test was used to assess deviations from the normal distribution. To evaluate changes between paired ordinal or metric parameters, the Wilcoxon signed rank sum test was applied. Paired changes in nominal variables with two or more classes were evaluated by the McNemar test. Differences in ordinal or metric variables between two or more independent groups were evaluated by the Mann–Whitney U test or by the Kruskal–Wallis test, respectively. For comparisons including more than two groups, post hoc pairwise comparisons were performed with Bonferroni correction for multiple testing.

Statistical analyses were performed in SPSS 27 software (IBM Corporation, Armonk, NY, USA). For all analyses, two-sided tests were performed, and *p* values of < 0.05 were considered statistically significant.

## Results

A total of 46 prostate cancer patients at a median age of 71 ± 8.0 years and a median disease duration of 41 months (range: 0–192) underwent analysis with both [^68^Ga]-Ga-PSMA-11 and [^18^F]-F-PSMA-1007. The median PSA value of all patients was 3.76 ng/ml (range: 0.3–113.7 ng/ml).

The PSA values of all patients ranged from 0.3 to 113.7 ng/ml (median: 3.76), while patients with BCR had lower values (median: 2.1; range: 0.3–11.1) than those with FU examination (median: 6.2; range: 4.1–40) and PS (median: 2.1; range: 13.4–113.7). Further patient characteristics are shown in detail in Table 1.

**Table 1:** Patients’ characteristics

Characteristics of 46 patients	Results
Age [years], mean, standard deviation	71, 6.9
PSA value [ng/ml], median, range	3.76, 0.32–113.7
Disease duration [months], median, range	41, 0–192
Days between [68Ga]-Ga-PSMA-11 and [18F]-F-PSMA-1007, mean, standard deviation, *median*	12, 8.0, *13*
Indication [*n*; %]	
Primary staging	10; 21.7%
Biochemical recurrence	30; 65.2%
After RPx	26 (86.7%)
After RTx/Brachytherapy	4 (13.3%)
Follow-up/PRLT-evaluation	6; 13.0%

Gleason score (GSC)	*n*
6	2 (4.3%)
7	20 (43.5%)
8	6 (13.0%)
9	18 (39.1%)
10	0

RPx, radical prostatectomy; RTx, radiotherapy

### Indication

The patient group was quite heterogeneous: approximately two-thirds of the patients (*n* = 30; 65.2%) were referred to our department due to biochemically recurrent disease. Ten patients underwent examinations for primary staging (21.7%), and six patients underwent follow-up of known metastases or evaluation for PSMA-targeted radioligand therapy (PRLT) (13.0%). Figure 1 shows the indications for both PET devices.

### Normal tissue uptake

[^18^F]-F-PSMA-1007 showed higher normal tissue uptake, as expressed in SUV_mean,_ in the liver (13.0 vs. 7.0; *p* < 0.001) and spleen (13.2 vs. 8.7; *p* < 0.001) and to a lesser extent in the parotid glands (20.8 vs. 19.0; *p* = 0.002) than [^68^Ga]-Ga-PSMA-11. There were no differences in blood pool uptake (1.9 vs. 1,9; *p* = 0.93). The [^18^F]-F-PSMA-1007 uptake did not differ significantly between the liver and spleen (*p* = 0.615). However, the relevant reference tissue uptake for the miPSMA score was higher in the spleen for [^18^F]-F-PSMA-1007 than in the liver for [^68^Ga]-Ga-PSMA-11 (*p* < 0.001).

### miTNM staging

Both [^18^F]-F-PSMA-1007 and [^68^Ga]-Ga-PSMA-11 PET were evaluated independently in terms of miTNM staging (Table 2).

**Table 2:** miTNM stages by tracer

miTNM stage	[^18^F]-F-PSMA-1007 [*n*]	[^68^Ga]-Ga-PSMA-11 [*n*]
T		
T0	26 (56.5%)	26 (56.5%)
R	7 (15.2%)	7 (15.2%)
T2u	**5 (10.9%)**	**4 (8.7%)**
T2m	**3 (6.5%)**	**4 (8.7%)**
T3a	1 (2.2%)	1 (2.2%)
T3b	2 (4.3%)	2 (4.3%)
T4	2 (4.3%)	2 (4.3%)
N		
N0	32 (69.6%)	32 (69.6%)
N1	**7 (15.2%)**	**10 (21.7%)**
N2	**7 (15.2%)**	**4 (8.7%)**
M		
0	27 (58.7%)	28 (60.9%)
1a	4 (8.7%)	4 (8.7%)
1b	12 (26.1%)	11 (23.9%)
1c	3 (6.5%)	3 (6.5%)

T2u (unifocal) T2m (multifocal)

Differences between both tracers were marked in bold

Different miTNM stages were obtained in nine different patients (19.6%), most commonly in miT staging (five patients). None of the differently staged patients were under active androgen-deprivation therapy (ADT). Further clinical details of all patients with disconcordant miTNM stages are listed in Table 3.

**Table 3:** Patients with disconcordant miTNM stages

ΔmiTNM	Indication	ADT	GSC	RPx	RTX	PSA	Device	[^18^F]-F-PSMA-1007	[^68^Ga]-Ga-PSMA-11	miTNM
T	BCR	Naive	6	No	Seeds	4.99	PET/CT	**T0**	TR	N0M0
	PS	Naive	8	No	No	13.80	PET/CT	**T2u**	T2m	N0M0
	BCR	Naive	9	Yes	No	1.98	PET/CT	**TR**	T0	N0M0
	BCR	Ended	7	Yes	Yes	2.04	PET/CT	**TR**	T0	N1M1a
	BCR	Naive	9	Yes	No	0.63	PET/CT	T0	**TR**	N1M0
N	BCR	Naive	7	Yes	Yes	3.09	PET/CT	**N2**	N1	TRM1c
	FU	Naive	7	Yes	No	4.40	PET/CT	**N2**	N1	T0M0
	PS	Naive	7	No	No	113.70	PET/CT	**N2**	N1	T3bM0
M	BCR	Naive	9	Yes	No	0.58	PET/CT	**M1b**	M0	T0N1

ADT, androgen depriving therapy; GSC, Gleason Score, RPx, radical prostatectomy RTx, radiotherapy, BCR, biochemical recurrence; PS, primary staging; FU, follow-up; T2u (unifocal) T2m (multifocal), TR (local recurrence). Clinical consensus was made on the bold stage

### miT staging

Different miT stages were obtained in only 5 (10.9%) of the 46 patients (Table 4).

**Table 4:** miT stage by tracer

miT stage		[^18^F]-F-PSMA-1007 [*n*]							
		0	2m	2u	3a	3b	4	R	Σ
[^68^Ga]-Ga-PSMA-11	0	24	0	0	0	0	0	2	26
	2m	0	3	1	0	0	0	0	4
	2u	0	0	4	0	0	0	0	4
	3a	0	0	0	1	0	0	0	1
	3b	0	0	0	0	2	0	0	2
	4	0	0	0	0	0	2	0	2
	R	2	0	0	0	0	0	5	7
	Σ	26	3	5	1	2	2	7	46

Four of these different miT stages occurred in BCR patients, and only one occurred in a PS patient.

**Fig. 2:**
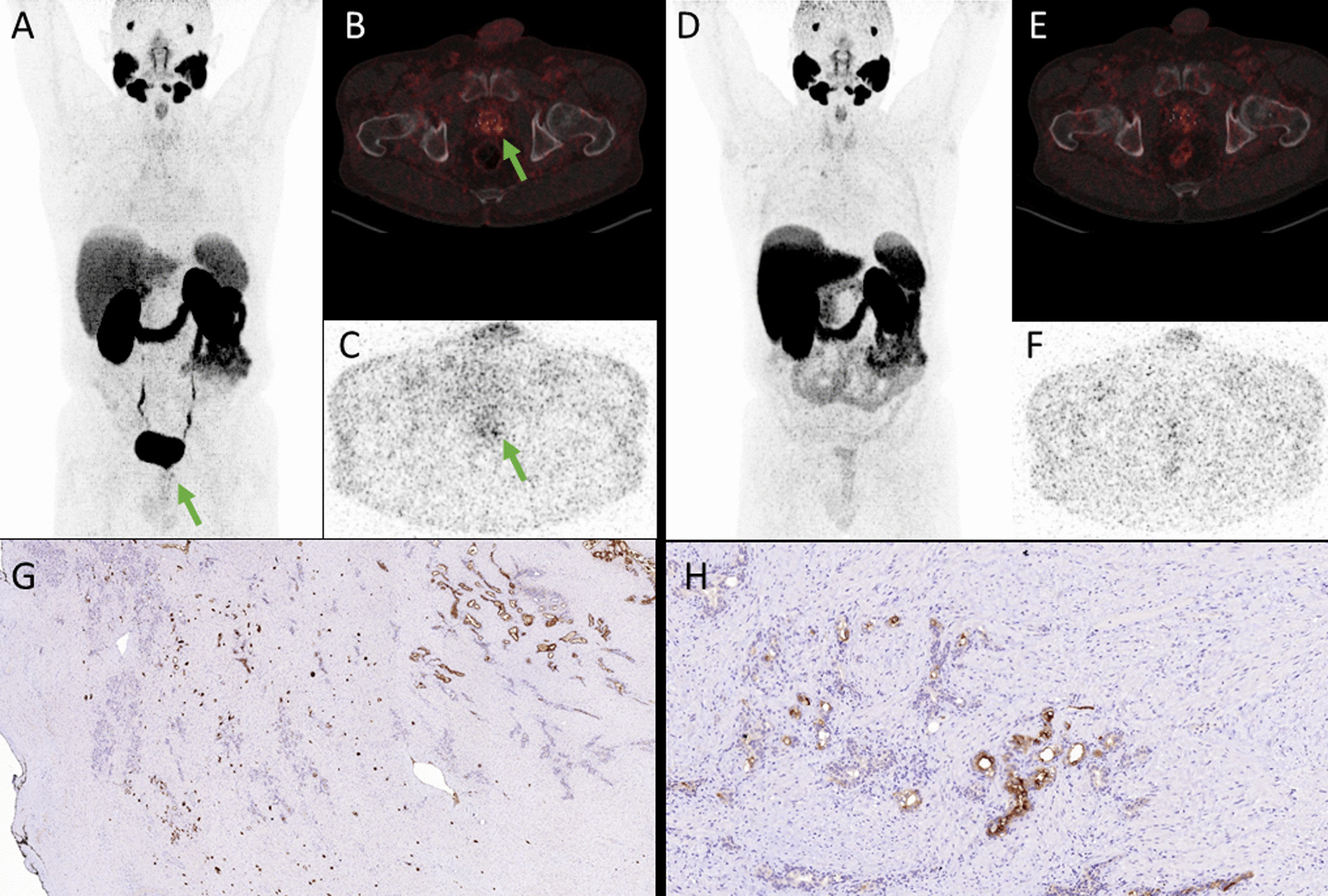
MIP of [^68^Ga]-Ga-PSMA-11 PET (**A**) with marginally positive (SUV_max_ 7.7) oligofocal local recurrence after seed implantation in both the right and left dorsoapical prostate lobe (green arrow). The accumulation was retrievable in both the attenuation corrected fused axial slices (**B**) and in the non-attenuation corrected axial slices (**C**). In [^18^F]-F-PSMA-1007 PET neither in the MIP (**D**) nor in the attenuation corrected fused axial slices (**E**) nor in the non-attenuation corrected axial slices (**F**) focal uptake was noted. Immunhistochemical images (**G**, **H**) show several, diffuse, small tumor cell nests with moderate to intense PSMA-expression

Histological confirmation after radical prostatectomy (RPx) was conducted in only one case with multifocal local recurrence after seed implantation, which was exclusively but only discretely detectable in [^68^Ga]-Ga-PSMA-11 PET (Fig. 2).

**Fig. 3:**
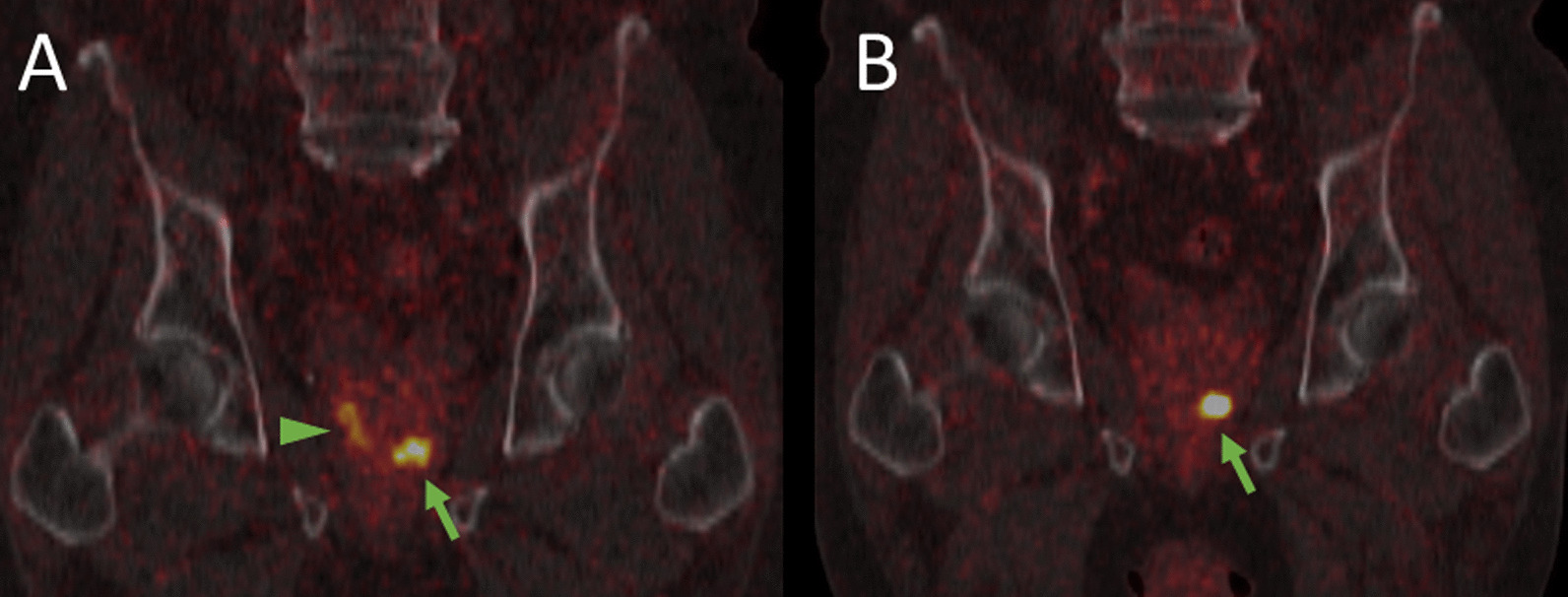
Coronal fused [^68^Ga]-Ga-PSMA-11 (**A**) and [^18^F]-F-PSMA-1007 PET/CT (**B**). Concordant focal uptake in the primary tumor in the left apical lobe (green arrow). The longitudinal uptake in the peripheral lobe in the [^68^Ga]-Ga-PSMA-11 PET/CT (arrowhead) finds no counterpart in [^18^F]-F-PSMA-1007 PET/CT

In a single primarily staged patient, there was only unifocal left-sided tracer uptake in [^18^F]-F-PSMA-1007 PET, while in [^68^Ga]-Ga-PSMA-11 PET, there was additional peripheral uptake in the right peripheral lobe (Fig. 3). As the patient underwent primary radiation therapy, no histologic confirmation of the whole prostate was conducted. However, the prostate biopsy was only positive in the left lobe.

**Fig. 4:**
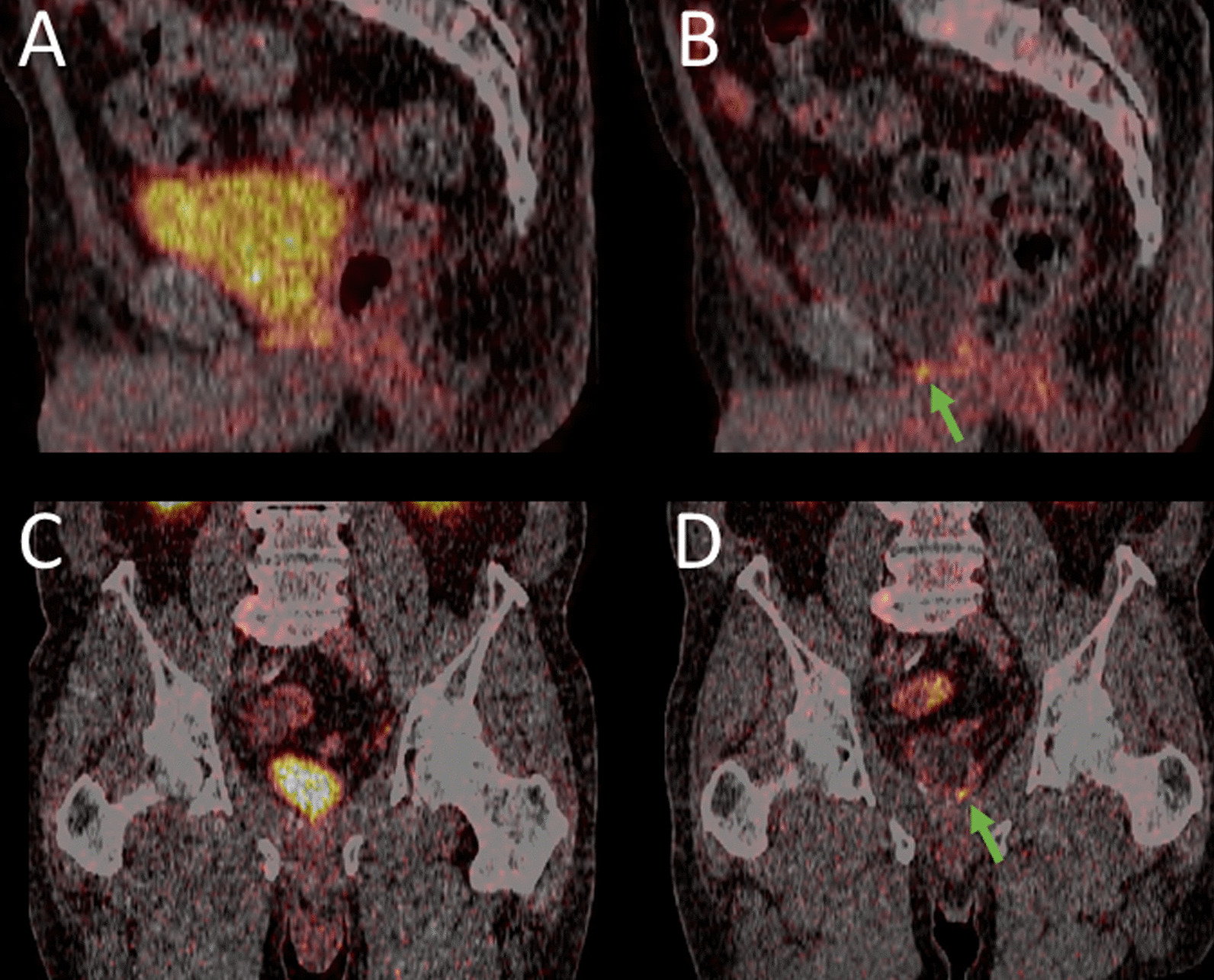
Sagittal fused [^68^Ga]-Ga-PSMA-11 (**A**) and [^18^F]-F-PSMA-1007 PET/CT (**B**) and coronal fused [^68^Ga]-Ga-PSMA-11 (**C**) and [^18^F]-F-PSMA-1007 PET/CT (**D**). While in [^68^Ga]-Ga-PSMA-11, there is only homogenous uptake in the prostatic fossa (A) clear focal uptake indicates local tumor recurrence in [^18^F]-F-PSMA-1007 PET/CT (green arrow)

In two other patients, local recurrence was only detected by [^18^F]-F-PSMA-1007 PET, and consecutive salvage radiotherapy (RTx) and ADT were initiated (Fig. 4).

**Fig. 5:**
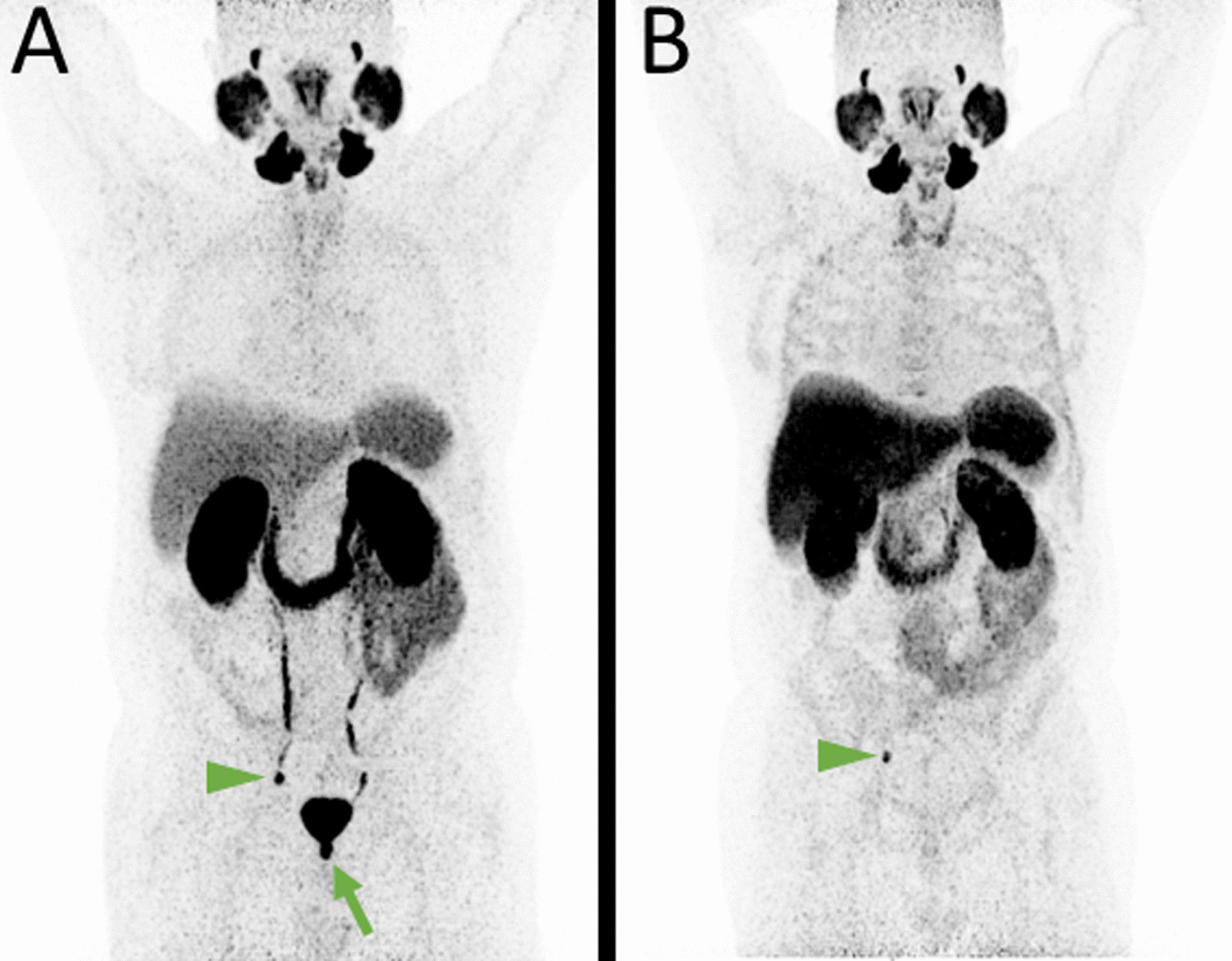
MIP of [^68^Ga]-Ga-PSMA-11 (**A**) and [^18^F]-F-PSMA-1007 PET (**B**). Only in the [^68^Ga]-Ga-PSMA-11 PET, intense uptake in the prostatic fossa is noteable (green arrow). The absence of any uptake in [^18^F]-F-PSMA-1007 PET suggests the presence of urinary retention. Concordantly present external iliac lymph node metastasis on the right (arrow heads). However, in [^18^F]-F-PSMA-1007 PET due to missing ureteral activity, the demarcation is much better. Auxiliary finding in **B** Disseminated presumable unspecific uptake in the skeleton and moderate thyreoidal uptake

In the fifth patient with BCR, in addition to concordantly detected pelvic lymph node metastasis, [^68^Ga]-Ga-PSMA-11 PET was suspicious for local tumor recurrence, which could be attributed to urinary retention after negativity in [^18^F]-F-PSMA-1007 PET (Fig. 5), and ADT was initiated.

In the 22 patients with tracer uptake in the prostate or in the prostatic bed in at least one study, the SUV_max_ of [^18^F]-F-PSMA-1007 was higher in 13 patients, while the mean did not differ significantly (*p* = 0.961). The SUV_peak_ of [^18^F]-F-PSMA-1007, however, was only higher in eight patients, while the SUV_mean_ did not differ significantly (*p* = 0.961). The miPSMA score, with different reference tissues in both tracers, was identical in 13 patients; only three patients had a higher prostatic miPSMA score in [^18^F]-F-PSMA-1007, while six had a lower miPSMA score.

The median SUV_max_ (31.5 vs. 32.7; *p* = 0.658) and SUV_peak_ (7.0 vs. 8.0; *p* = 0.158) of the main lesions did not differ significantly between [^18^F]-F-PSMA-1007 and [^68^Ga]-Ga-PSMA-11.

### miN staging

The MiN0 stage was concordantly indicated by both tracers in all 32 patients (69.6%) (Table 5).

**Table 5:** miN stage by tracer

miN stage		[^18^F]-F-PSMA-1007 [*n*]			
		0	1	2	Σ
[^68^Ga]-Ga-PSMA-11	0	32	0	0	32
	1	0	7	3	10
	2	0	0	4	4
	Σ	32	7	7	46

Regional lymph node metastases were consistently present in the same 14 of the 46 patients (30.4%). Eighteen of 46 (39.1%) patients had pelvic and/or extrapelvic lymph node metastases. The 14 patients with pelvic lymph node metastases had these in a total of 31 pelvic regions in [^18^F]-F-PSMA-1007 PET and in a little lesser 28 regions (15 × unifocal, 7 × oligofocal, 6 × multifocal) in [^68^Ga]-Ga-PSMA-11 PET. [^18^F]-F-PSMA-1007 indicated more lesions than [^68^Ga]-Ga-PSMA-11 in five regions (3 × external iliac, internal iliac and common iliac), while the opposite was the case only in one internal iliac region (Table 6).

**Table 6:** Lymphonodal uptake by region

Lymph node region	[^18^F]-F-PSMA-1007 [*n*] (uni/oligo/multi)	[^68^Ga]-Ga-PSMA-11 [*n*] (uni/oligo/multi)	F +	Ga +
Internal iliac right	3 (2/1/0)	3 (3/0/0)	1	0
Internal iliac left	4 (3/1/0)	5 (4/1/0)	0	1
External iliac right	5 (2/2/1)	4 (2/1/1)	1	0
External iliac left	5 (3/1/1)	3 (2/0/1)	2	0
Common iliac right	1 (1/0/0)	1 (1/0/0)	0	0
Common iliac left	3 (3/0/0)	2 (2/0/0)	1	0
Obturator right	1 (0/1/0)	1 (0/1/0)	0	0
Obturator left	1 (0/1/0)	1 (0/1/0)	0	0
Presacral	5 (1/2/2)	5 (1/2/2)	0	0
Other pelvic	3 (0/1/2)	3 (0/1/2)	0	0
Σ regional	31 (15/10/6)	28 (15/7/6)	5	1
Retroperitoneal	5 (1/2/2)	4 (0/2/2)	1	0
Other extrapelvic	6 (4/1/1)	6 (4/1/1)	0	0

Differences in terms of miN staging were obtained in only three patients (6.5%) and only between miN1 and miN2 (Table 2). In all cases, [^18^F]-F-PSMA-1007 PET led to upstaging from miN1 to miN2, while downstaging was not observed.

However, four of the 32 miN0 patients had exclusively extrapelvic lymph node metastases in both studies. Table 6 shows the pattern of lymph node metastases by region and tracer. The manifestations were balanced within the pelvic regions, and no clear predilection spot could be identified.

**Fig. 6:**
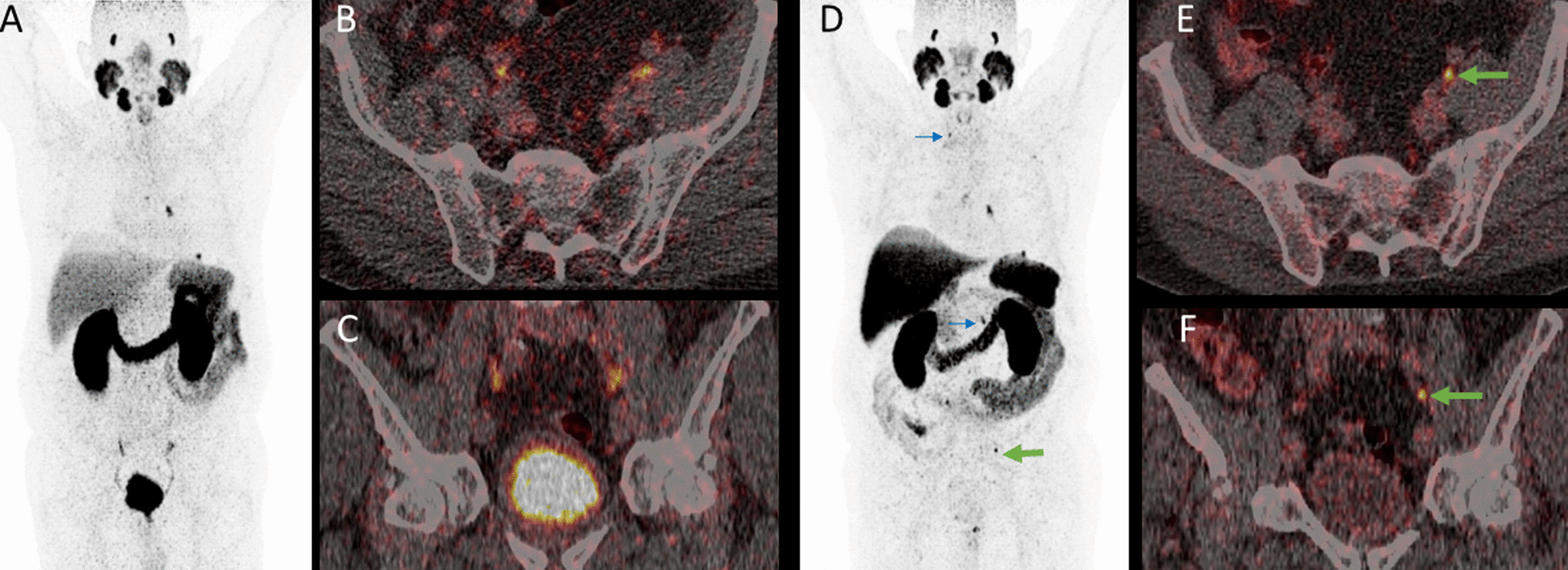
MIP of [^68^Ga]-Ga-PSMA-11 PET (**A**) with quite similar longitudinal uptake in both ureters. In both axial (**B**) and coronal fused (**C**) PET/CT images, an accessory paraureteral lesion is not detectable. In [^18^F]-F-PSMA-1007 PET, the paraureteral iliac extern lymph node metastasis is clearly depictable in both the MIP (**D**) and the axial (**E**) and coronal fused (**F**) PET/CT images (green arrow). Please note several other concordant malignancy-associated findings: local tumor recurrence in the left prostatic lobe, infracarinal and left-hilar lymph node metastases. Additionally, there is a small focus in the left lower lobe of the lung (in detail in Fig. 7). Auxiliary findings: presumable unspecific uptake in cervical and coeliac ganglia (blue arrow) in [^18^F]-F-PSMA-1007 PET/CT. Unspecific ileocoecal uptake is also present in [^18^F]-F-PSMA-1007 PET

**Fig. 7:**
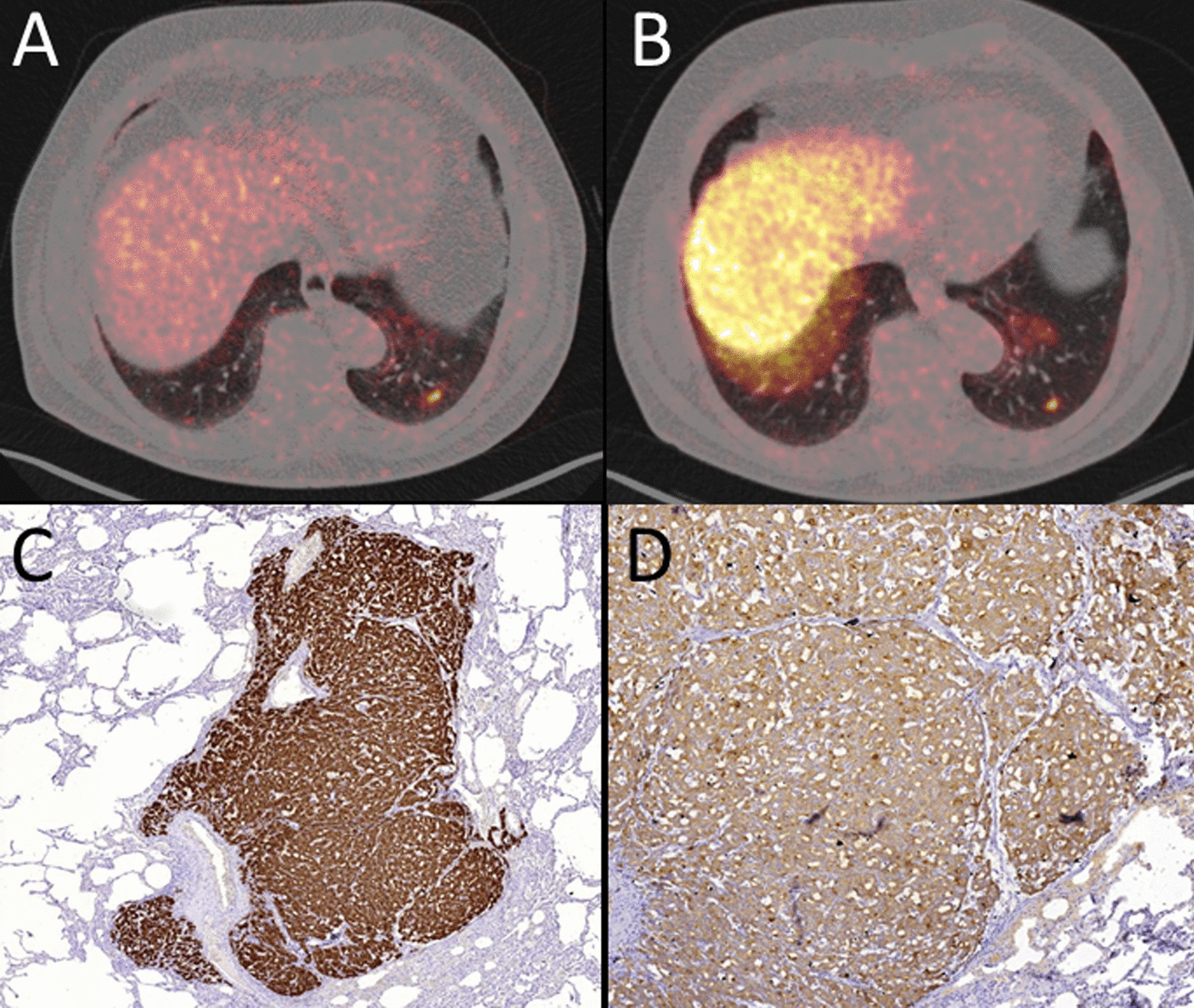
Axial fused [^68^Ga]-Ga-PSMA-11 PET/CT (**A**) and [^18^F]-F-PSMA-1007 (**B**) with concordantly intense focal uptake in a pulmonary metastasis in the left lower pulmonary lobe. Immunhistochemial confirmation of PSMA- (**C**) and PSA- (**D**) expression of the pulmonary metastasis (**D**)

Extrapelvic lymph node metastases were noted in eight other patients (17.4%) in both [^18^F]-F-PSMA-1007 and [^68^Ga]-Ga-PSMA-11 PET. Most of these patients had retroperitoneal metastases (*n* = 5). Other, mostly additional locations were rare: supraclavicular (*n* = 1), hilar (*n* = 2), mediastinal (*n* = 1), axillary (*n* = 1) and inguinal (*n* = 1). In addition to his concordant hilar and mediastinal lymph node metastases and pulmonary metastasis, one patient had solitary paraureteral iliac lymph node metastasis solely detected by [^18^F]-F-PSMA-1007 (Figs. 6, 7).

The median SUV_max_ (28.9 vs. 24.9; *p* = 0.30) and SUV_peak_ (8.5 vs. 10.2; *p* = 0.08) of the most intense lymph node metastases did not differ significantly between [^18^F]-F-PSMA-1007 and [^68^Ga]-Ga-PSMA-11.

### miM Staging

The different tracer distributions between the two tracers in terms of distant metastases led to a different miM stage in only one patient (Table 3).

**Fig. 8:**
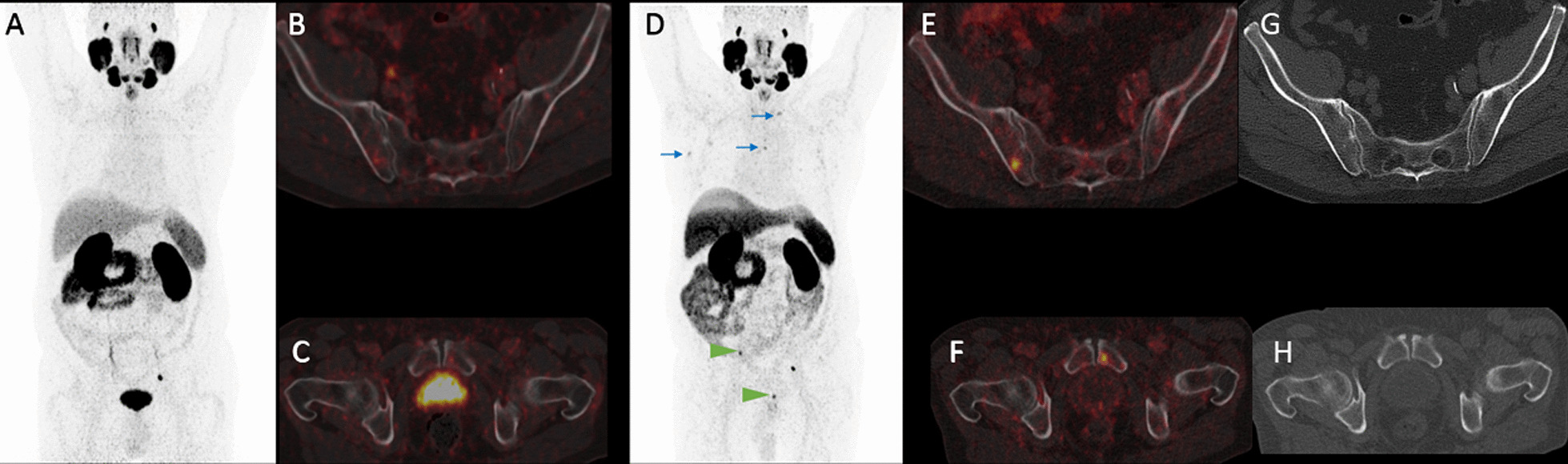
MIP of [^68^Ga]-Ga-PSMA-11 PET (**A**) with obvious focal uptake only paraureteral left. In axial fused PET/CT of the iliosacral region (**B**) and the symphysis (**C**), no focal uptake suggestive for malignancy is present. The MIP of [^18^F]-F-PSMA-1007 (**D**) shows beside the concordant paraureteral uptake on left side intensive focal uptake in the right iliac bone (SUV_max_: 16.5) and the left pubic bone (SUV_max_: 14.8 arrow heads). Axial fused PET/CT confirms the intraosseous location of both the iliac (**E**) and the pubic metastasis (**F**). While the right iliac lesion has a slight hypersclerotic correlate in CT-imaging (**G**), there is no morphologic correlate in the left pubic bone detectable in low-dose CT (**H**). Auxiliary findings in [^18^F]-F-PSMA-1007 (**D**): presumable unspecific uptake in cervical ganglia, mediastinal and axillary lymph nodes (blue arrows) as well as multifocally in the rib thorax

This patient with BCR had, in addition to a concordant solitary right external iliac lymph node metastasis, two bone metastases visualized only in [^18^F]-F-PSMA-1007 PET (Fig. 8).

The remaining patients were concordantly staged as miM0 (*n* = 27; 58.7%), miM1a (*n* = 4; 8.7%), miM1b (*n* = 8; 17.4%) or miM1c (*n* = 6; 13.0%).

In another patient, there was only a difference in the metastatic pattern, as [^18^F]-F-PSMA-1007 additionally indicated unifocal uptake in the [^68^Ga]-Ga-PSMA-11 oligofocal uptake.

Bone metastases were the most frequent manifestation of miM1. In [^18^F]-F-PSMA-1007 PET, 12 patients (26.1%) showed unifocal (*n* = 6), oligofocal (*n* = 3) or multifocal (*n* = 3) uptake in osseous metastases.

In [^68^Ga]-Ga-PSMA-11 PET, 11 patients (23.9%) showed unifocal (*n* = 7), oligofocal (*n* = 1) or multifocal (*n* = 3) uptake in osseous metastases. No patient had exclusive bone metastases on [^68^Ga]-Ga-PSMA-11 PET. Within the three patients with multifocal bone metastases, only concordant metastases occurred. Table 7 shows the pattern of metastatic bone lesions in both tracers.

**Table 7:** Bone metastatic pattern by tracer

Metastatic bone pattern		[^18^F]-F-PSMA-1007 [*n*]				
		0	Uni	Oligo	Multi	Σ
[^68^Ga]-Ga-PSMA-11	0	34	0	1	0	35
	Uni	0	6	1	0	7
	Oligo	0	0	1	0	1
	Multi	0	0	0	3	3
	Σ	34	6	3	3	46

The median SUV_max_ (22.9 vs. 27.6; *p* = 0.286) and SUV_peak_ (11.9 vs. 14.5; *p* = 0.286) of the most intense bone metastases did not differ significantly between [^18^F]-F-PSMA-1007 and [^68^Ga]-Ga-PSMA-11.

Pulmonary (*n* = 2) and soft tissue metastases (*n* = 1) were rare and were concordantly indicated in both studies. Figure 7 shows a histologically confirmed lung metastasis.

However, in one patient, in addition to a concordant PSMA-positive nodule, additional pulmonary metastasis was present in [^68^Ga]-Ga-PSMA-11 PET.

### Presumable unspecific lesions

Most likely, unspecific, discrete (miPSMA score 1) lymphonodal uptake was present in 24 (52.2%) of the [^18^F]-F-PSMA-1007 PET scans and in only 13 of the [^68^Ga]-Ga-PSMA-11 PET scans (28.2%).

**Fig. 9:**
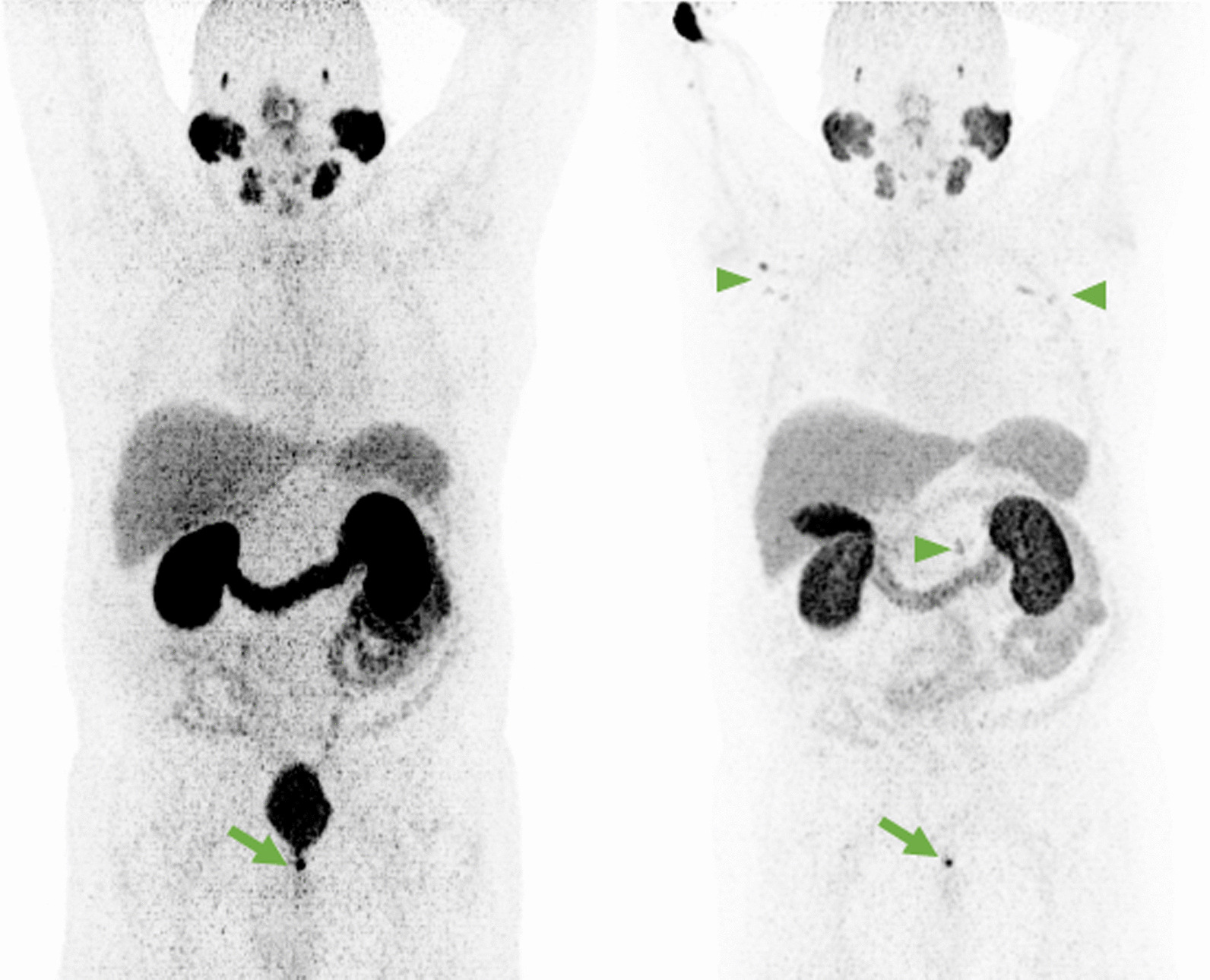
MIP of [^68^Ga]-Ga-PSMA-11 and [^18^F]-F-PSMA-1007 PET: Beside the concordant local recurrence in the left prostate bed (arrow), there is unspecific oligofocal uptake in the rip thorax, lymphonodally in the right axilla, the coeliac ganglia (arrow heads) and the gall bladder

[^18^F]-F-PSMA-1007 indicated (additional) exclusive presumable unspecific lymphonodal foci in 18 different patients (39.1%). While additional axillary lymphonodal uptake was noted in all 18 patients (39.1%), 6 patients had unspecific mediastinal/hilar uptake (13.0%), and 5 patients presented with unspecific auxiliary inguinal tracer accumulation. A patient with axillary lymph node uptake that was interpreted as unspecific is exemplarily shown in Fig. 9. In only one patient (2.2%) was there unretrieved uptake in an axillary lymph node (Table 8) compared to that analyzed with [^68^Ga]-Ga-PSMA-11.

**Table 8:** Prevalence of discordant presumable unspecific lesions

Type of presumable unspecific lesion		[^18^F]-F-PSMA-1007 [*n*]	[^68^Ga]-Ga-PSMA-11 [*n*]	*p*
Lymph node	Total (Σ; uni/oligo/multi)	24 (52.2%); 3/9/12	13 (28.3%); 0/10/3	< 0.001
	Total patients with more lesion	18 (39.1%)	1 (2.2%)	
	Axillary	18 (39.1%)	1 (2.2%)	
	Mediastinal/hilar	6 (13.0%)	–	
	Inguinal	5 (10.9%)	–	
Osseous	Total patients (Σ; uni/oligo/multi)	33 (71.7%); (4/7/22)	11 (23.9%); (5/6/0)	< 0.001
	Total patients with more lesion	28 (60.9%)		
	Total lesions/more lesions ribs	28/27 (58.7%)	6/0	< 0.001
	Total lesions/more lesions spine	15/11 (23.9%)	4/0	< 0.001
	Total lesions/more lesions pelvis	6/5 (10.9%)	1/0	0.025
	Total lesions/more lesions scapula	6/5 (10.9%)	1/0	0.025
	Total lesions/more lesions sternum	5/5 (10.9%)	0	0.025
	Total lesions/more lesions femur	2/2 (4.3%)	0	0.157
Ganglia	*Total patients with any ganglia uptake*	33 (71.7%)	20 (43.5%)	< 0.001
	Total lesions/more lesions cervical	30/13 (28.3%)	17/0	< 0.001
	Total lesions/more lesions coeliac	17/9 (19.6%)	8/0	< 0.001
	Total lesions/more lesions sacral	8/5 (10.9%)	3/0	0.025
Thyroid	*Total patients*	26 (56.5%)	4 (8.7%)	< 0.001
	Total patients with higher uptake	22		
Esophagus	*Total patients*	11 (23.9%)	24 (52.2%)	< 0.001
	Total patients with higher		13	
Ileocoecal	Total patients diffuse uptake	3 (6.5%)	1 (2.1%)	0.157
Pancreas	Total patients diffuse pancreatic uptake	2 (4.3%)	1 (2.1%)	0.317

Osseous uptake not suggestive of malignancy was quite rare in [^68^Ga]-Ga-PSMA-11 PET, with 11 patients (23.9%) presenting unifocal (10.9%) or oligofocal (13.0%) bone uptake without implication of malignancy. Neither multifocal presumable unspecific bone uptake nor exclusive lesions were present.

In [^18^F]-F-PSMA-1007 PET, a large majority (71.7%, *n* = 33) of the patients featured a low-intensity (miPSMA score 1) bone uptake, which was unifocal (8.7%), oligofocal (15.2%) or mostly multifocal (47.8%) and unsuspicious for malignant origin.

[^18^F]-F-PSMA-1007 PET indicated exclusive osseous lesions in 28 patients (60.9%). Twenty-seven of those patients had additional uptake in the ribs (58.7% of all patients). As shown in Table 8, other locations with discordant bone uptake in [^18^F]-F-PSMA-1007 were the spine (23.9%), pelvis (10.9%) and scapula (10.9%). Figure 9 shows a patient with additional oligofocal rib lesions in [^18^F]-F-PSMA-1007 PET that were interpreted as unspecific.

Small focal uptake in the cervical, coeliac and sacral ganglia was present more often in the [^18^F]-F-PSMA-1007 studies (71.8% vs. 43.5%). The cervical ganglia were affected in all 13 cases (28.3%) with additional ganglionic uptake in [^18^F]-F-PSMA-1007 PET. Exclusive tracer uptake in the coeliac (19.6%) and presacral ganglia (10.9%) was detected less frequently and always in addition to cervical ganglia uptake.

Homogeneous esophageal uptake that was not suspicious for malignancy was more common in [^68^Ga]-Ga-PSMA-11 PET than in [^18^F]-F-PSMA-1007 PET (52.2% vs. 23.9%; *p* < 0.001), while uptake in the thyroid gland occurred almost exclusively with [^18^F]-F-PSMA-1007 (56.4% vs. 8.7%; *p* < 0.001).

**Fig. 10:**
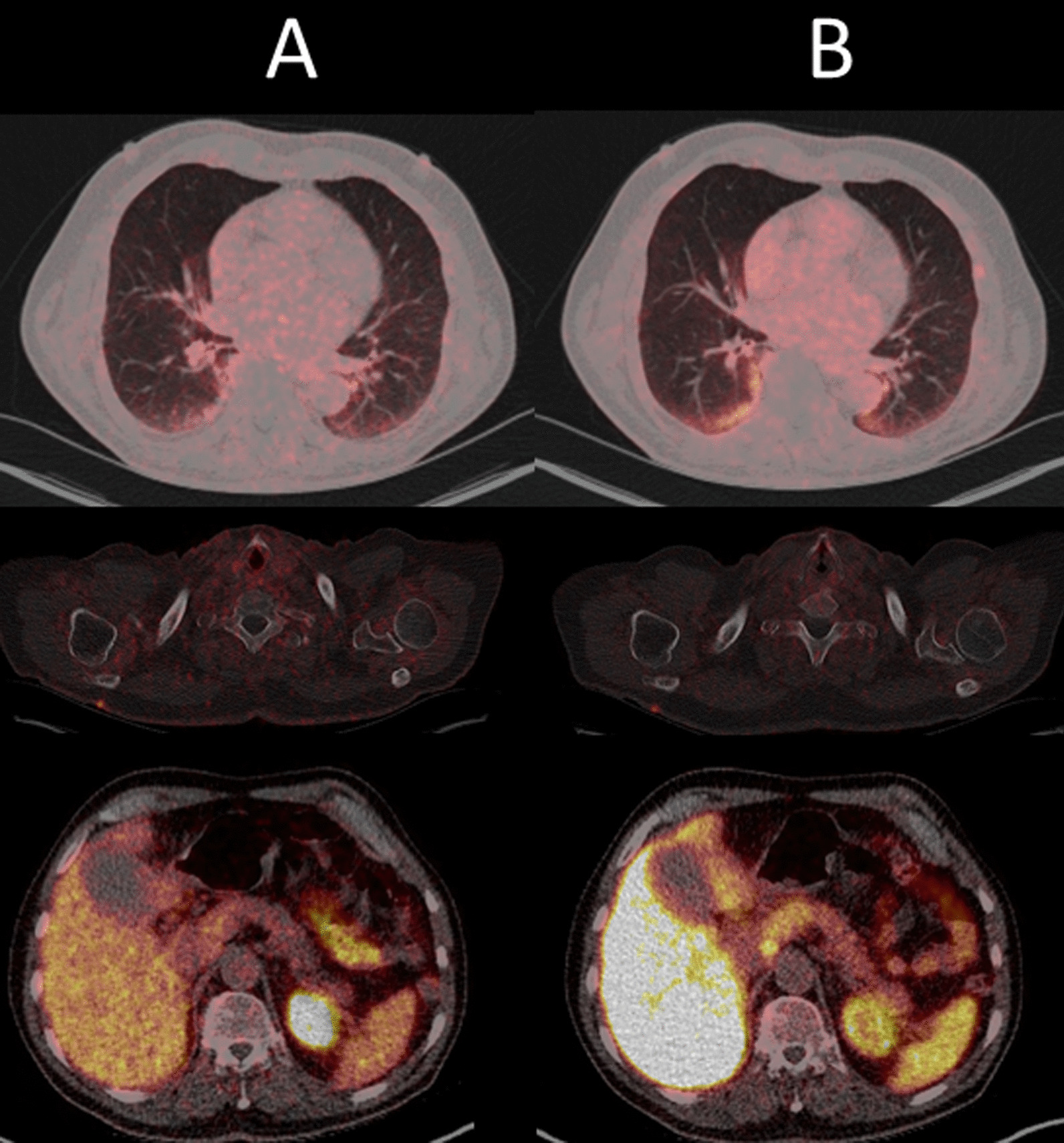
Axial fused PET/CT with concordantly presumable unspecific [^68^Ga]-Ga-PSMA-11 (**A**) and [^18^F]-F-PSMA-1007 PET/CT (**B**) uptake in bipulmonary infiltrations, as well as in a cutaneous lesion in the right dorsal thoracic wall, clinically evaluated as keloid scar

Two patients concordantly presented homogenous discrete pancreatic uptake, and one patient had unspecific uptake in a cutaneous keloid scar. Another patient had diffuse low-intensity (miPSMA score 1) uptake in a pneumonic infiltration. Figure 10 shows an example of presumably unspecific uptake patterns.

In [^18^F]-F-PSMA-1007 PET, a device-dependent difference in presumable unspecific uptakes was observed between PET/CT and PET/MRI. Non-malignancy-associated uptake in bones and ganglia (77.5% vs. 33.3%; *p* = 0.027 in both locations) occurred more frequently or even exclusively, as in lymph nodes (60.0% vs. 0%; *p* = 0.007), on PET/CT. As shown in Table 9, significant differences could not be shown for [^68^Ga]-Ga-PSMA-11 PET.

**Table 9:** Presumable unspecific uptake in lymph nodes, bones and ganglia by device

Presumable unspecific uptake		PET/CT [*n*]/(% of 40 PET/CT patients)	PET/MR [*n*]/(% of 6 PET/MR patients)	*p* value
Lymph nodes	[^18^F]-F-PSMA-1007	24 (60.0%)	0	0.007
	[^68^Ga]-Ga-PSMA-11	13 (32.5%)	0	0.103
Bones	[^18^F]-F-PSMA-1007	31 (77.5%)	2 (33.3%)	0.027
	[^68^Ga]-Ga-PSMA-11	10 (25.0%)	1 (16.7%)	0.659
Ganglia	[^18^F]-F-PSMA-1007	31 (77.5%)	2 (33.3%)	0.027
	[^68^Ga]-Ga-PSMA-11	19 (47.5%)	1 (16.7%)	0.160

## Discussion

The different biodistributions of [^68^Ga]-Ga-PSMA-11 and [^18^F]-F-PSMA-1007 led to disconcordant tracer accumulations, especially in lesions of unspecific/benign origin and to a far lesser extent in prostate cancer manifestations, which is in line with the previously published head-to-head [17] and matched comparisons [16].

The far lower urinary tracer excretion of [^18^F]-F-PSMA-1007 may lead to superiority against [^68^Ga]-Ga-PSMA-11 [24] and renally excreted PSMA tracers in general [25] in terms of evaluation of local tumor recurrence and, possibly, in primary tumor detection. However, alternate biliary excretion may veil hepatic and neighboring tumor manifestations.

Kuten et al. [17] showed an almost perfect concordance between [^18^F]-F-PSMA-1007 and [^68^Ga]-Ga-PSMA-11 in terms of identifying intermediate- and high-risk prostate cancer manifestations in primary staging, with [^18^F]-F-PSMA-1007 indicating additional low-grade lesions. In accordance, miT staging was based on concordant lesions in nearly 90% of patients. Histologically confirmed exclusive lesions (after RPx or biopsy) were detected for each tracer once, implicating that there is no systematic advantage of one tracer in terms of primary staging. No significant difference in terms of SUV_max_ or SUV_peak_ was noted.

Even though not relevantly advantageous in terms of general miTNM staging, [^18^F]-F-PSMA-1007 might be superior in terms of recurrence detection [24, 26] and in exact tumor delineation, particularly for radiotherapy planning [27], especially if a local tumor boost is intended [28, 29] due to the absence of bladder activity and, to a far lesser extent, its higher spatial resolution. The disadvantage of [^68^Ga]-Ga-PSMA-11 could be mitigated by diuretic premedication [30, 31]. Interestingly, as shown in Fig. 2, there are, even though rarely, cases in which intraprostatic tumor burdens are exclusively indicated by [^68^Ga]-Ga-PSMA-11. An explanation therefore might be found either in the different PSMA affinities or, ironically, in the lower spatial resolution of [^68^Ga]-Ga-PSMA-11. Lower resolution might be a reason for two or more small adjacent tumor nests to be recognized as one focus in [^68^Ga]-Ga-PSMA-11, while they are obscured in [^18^F]-F-PSMA-1007 as simple heterogeneity.

Differences in the detectability of lymph node metastases may occur both because of different total uptake of the lesion and because of altered levels of the local tumor background, which may be higher in the pelvis in biliary excreted [^18^F]-F-PSMA-1007 [26]. As the reference tissue uptake for miPSMA scoring in the liver in [^68^Ga]-Ga-PSMA-11 PET and the spleen in [^18^F]-F-PSMA-1007 PET were significantly different, no further lesion-based comparisons were performed based on scoring. Intrapelvic lymph node metastases were concordantly excluded in nearly 70% of all patients (N0 situation).

Thus, differences in miN stages occurred only in the number of metastases within one region or the number of regions harboring lymph node metastases. A region-based evaluation of the 14 patients with concordant miN1 or miN2 revealed only 5 additionally infested lymph node regions in [^18^F]-F-PSMA-1007 PET compared with [^68^Ga]-Ga-PSMA-11 PET, while the opposite was the case in just one site. An explicit region-based analysis could not identify a predilection spot of superiority. In addition to the not explicitly evaluated tumor-background ratio (TBR), a potential locoregional diagnostic advantage of biliary excreted [^18^F]-F-PSMA-1007 could not be shown, as both SUV_max_ and SUV_peak_ were not significantly different. Patient-based differences were even rarer. In [^18^F]-F-PSMA-1007 PET of three patients, the number of infested lymph node regions increased, leading to an upstaging from N1 to N2. From a clinical point of view, these differences did not influence further therapeutic management.

For extrapelvic lymph node manifestations, all lesions were indicated concordantly by both tracers. The miM staging was shown to be even more concordant (97.8%) between the two tracers than the miT and miN staging. [^18^F]-F-PSMA-1007 indicated more-predominant or exclusive bone metastases in one patient each. Thus, only in one patient who had BCR and concordantly indicated pelvic lymph node metastasis did [^18^F]-F-PSMA-1007 PET indicate a higher miM stage. No tracer outperformed the other in terms of SUV_max_ and SUV_peak_. The few detected PSMA-positive pulmonary and soft tissue metastases were focally consistently indicated by both tracers.

Thus, especially in terms of miN- and miM staging, [^18^F]-F-PSMA-1007 and [^68^Ga]-Ga-PSMA-11 can be considered exchangeable [15].

In concordance with the results of a matched tracer comparison of biochemically recurrent PCa patients by Rauscher et al., unspecific tracer accumulations in previously published predilection points [22] occurred intraindividually far more frequently in [^18^F]-F-PSMA-1007 PET [16].

Multifocal low-intensity bone uptake, especially in the ribs, without correlation in morphologic imaging, was the most common manifestation of unspecific uptake in [^18^F]-F-PSMA-1007 PET. This pattern is well known [16, 25, 32], although it has not yet been fully explained, and rarely represents a diagnostic challenge in many cases. Recently, Arnfield et al. suggested an SUV_max_ threshold of 7.2 under which these lesions could be interpreted as likely benign [33]. However, the long-term impact of these presumable unspecific osseous foci in [18F]-F-PSMA-1007 PET has yet to be discovered.

Non-malignant uptake in ganglia is also well known for both [^68^Ga]-Ga-based [22, 34–36] and [^18^F]-F-based ligands [16, 37] with different intensities and patterns [38]. Awareness of ganglia uptake and careful examination and evaluation within the clinical context allows in most cases differentiation against metastatic lymph node uptake [39]. Unspecific uptake due to neoangiogenesis occurred in both studies, similar to patients with a keloid scar [40].

The high rate of tracer accumulation attributed to benign genesis is already known for [^68^Ga]-Ga-PSMA-11 [41, 42] and seems to be even higher for [^18^F]-F-PSMA-1007. One possible explanation of the higher SUV_mean_ in the liver, spleen and parotid glands evaluated with [^18^F]-F-PSMA-1007 compared to [^68^Ga]-Ga-PSMA-11 is the higher spatial resolution of ^18^F compared with ^68^Ga as a result of the lower end-point positron energy. This may have caused a more focal impression in [^18^F]-F-PSMA-1007 images. We will further investigate in detail the individual influence of the physical properties of the nuclide and the reconstruction process on SUV values, image quality and spatial resolution.

In addition to these measurement factors and biokinetic aspects, another explanation focuses on the PSMA ligand itself, which has been shown to have different affinities for different tissues [8, 43, 44], as in our analysis non-malignancy associated uptake in the thyroid gland was more common with [^18^F]-F-PSMA-1007, and homogenous esophageal uptake was more common with [^68^Ga]-Ga-PSMA-11.

The study has several limitations resulting from its retrospective design, especially in terms of the heterogeneous patient group, as it was recruited consecutively from routine clinical practice. The majority of patients were examined due to BCR and early tumor stages. For this reason, the tumor localization in the prostate bed and in pelvic lymph nodes was incidence-related and of higher relevance than the visualization of visceral (especially hepatic) metastases. This setting, even though representative for routine clinical practice, might have been advantageous for analysis by [^18^F]-F-PSMA-1007. As clinical stages progress, the advantages of [^18^F]-F-PSMA-1007 over [^68^Ga]-Ga-PSMA-11 possibly fade, as the exact identification of all intrapelvic regions loses increasing clinical and therapeutic relevance. In terms of the evaluation of PRLT in Stage IV patients, [^68^Ga]-Ga-PSMA-11 is possibly even superior, as its biodistribution is closer to that of [^177^Lu]-Ga-PSMA-617 than to that of [^18^F]-F-PSMA-1007 [8, 14, 45]. The differences between [18F]-F-PSMA-1007 and [^68^Ga]-Ga-PSMA-11 in PRLT evaluation, however, will rarely yield clinical consequences, as both tracers will mostly consensually lead to an indication for PRLT, regardless of the exact number of PSMA-positive lesions. In the oligometastatic setting, these differences are of higher importance, as exclusively indicated lymphonodal or osseous tumor manifestations have therapeutic implications [46–49].

Histological confirmation was strived for only if clinically necessary. As all patients received both examinations on the same device, the degree of influence of the detector and the algorithm could not be further attributed at this point.

Another conceivable limitation is a certain variability of PSMA ligand accumulation between days. However, olde Heuvel et al. demonstrated the clinical irrelevance of this diurnal variability, at least for [^68^Ga]-Ga-PSMA-11 [50].

## Conclusion

In our retrospective head-to-head comparison, the radiotracers [^18^F]-F-PSMA-1007 and [^68^Ga]-Ga-PSMA-11 were, regardless of the indication, widely exchangeable in terms of metabolic TNM staging of prostate cancer.

However, there are assumptions that [^18^F]-F-PSMA-1007 indicates more presumable unspecific lesions than [^68^Ga]-Ga-PSMA-11 but is advantageous in terms of tumor delineation. A significant incremental value for a routinely performed two-tracer study could not be shown. As it seems for most nuclear medicine departments to be at least challenging to provide both [^18^F]-F-PSMA-1007 and [^68^Ga]-Ga-PSMA-11 PET, it is reasonable to choose the PSMA radiotracer depending on local availability with attention to the greater occurrence of nonspecific bone findings with [^18^F]-F-PSMA-1007.

## Data Availability

Anonymized data can be offered upon request.

## Abbreviations

ADT: Androgen deprivation therapy
BCR: Biochemically recurrence
CT: Computed tomography
[^18^F]-F-PSMA-1007: [^18^F]-F-PSMA-1007-prostate-specific membrane antigen 1007
FU: Follow-up
[^68^Ga]-Ga-PSMA-11: [^68^Ga]Ga-prostate-specific membrane antigen 11
GSC: Gleason score
miPSMA score: Molecular imaging prostate-specific membrane antigen score
miTNM stage: Molecular imaging TNM stage
MRI: Magnetic resonance imaging
OSEM: Ordered subset expectation maximization
PCa: Prostate cancer
PET: Positron emission tomography
PRLT: PSMA radio-ligand therapy
PS: Primary staging
PSA: Prostate-specific antigen
PSF: Point spread function
PSMA: Prostate-specific membrane antigen
RPx: Radical prostatectomy
RTx: Radiotherapy
SUV_max_: Maximum standard uptake value
SUV_mean_: Mean standard uptake value
SUV_peak_: Peak standard uptake value
TBR: Tumor to background ratio
TOF: Time-of-flight
VOI: Volume of interest
